# Changes in choroidal thickness in patients with diabetic retinopathy

**DOI:** 10.1007/s10792-017-0459-9

**Published:** 2017-02-13

**Authors:** Zaigen Ohara, Hitoshi Tabuchi, Shunsuke Nakakura, Yuki Yoshizumi, Hitomi Sumino, Yukiko Maeda, Yoshiaki Kiuchi

**Affiliations:** 1 Department of Ophthalmology, Saneikai Tsukazaki Hospital, 68-1 Aboshiku Waku, Himeji, 671-1227 Japan; 2 Department of Ophthalmology and Visual Sciences, Graduate School of Biomedical Sciences, Hiroshima University, Hiroshima, Japan

**Keywords:** Diabetic retinopathy, Panretinal photocoagulation, Choroidal thickness, Swept-source optical coherence tomography

## Abstract

**Purpose:**

To investigate the changes in choroidal thickness (ChT) following panretinal photocoagulation (PRP) for diabetic retinopathy (DR) and compare ChT in relation to DR severity.

**Methods:**

Thirty-two eyes [19 eyes with proliferative DR (PDR) and 13 eyes with severe nonproliferative DR (NPDR)] for which PRP was necessary were analyzed. ChT was measured before PRP and at 1, 3, and 6 months after PRP using the swept-source optical coherence tomography. ChT of the 61 eyes matched with the PDR patients for the mean age and axial length was also measured and statistically compared in relation to severity.

**Results:**

The central field ChT before PRP treatment was 268.6 ± 104.5 µm (mean ± standard deviation) and was significantly decreased at 1, 3, and 6 months after PRP (254.5 ± 105.3, 254.2 ± 108.2, and 248.1 ± 101.8 µm, respectively, *P* < 0.0001). The central field ChT of severe NPDR (323.2 ± 61.3 µm) was significantly thicker than that of normal (248.3 ± 70.7 µm) and mild to moderate NPDR (230.0 ± 70.3 µm, *P* = 0.0455 and 0.0099, respectively). Moreover, the central field ChT of PDR (307.3 ± 84.1 µm) was significantly thicker than of mild to moderate NPDR (*P* = 0.0169).

**Conclusion:**

ChT significantly decreased after PRP, which continued for at least 6 months after treatment. ChT of severe NPDR and PDR was significantly thicker than that of mild to moderate NPDR. ChT of patients with DR was changed according to the treatment and severity of DR.

**Electronic supplementary material:**

The online version of this article (doi:10.1007/s10792-017-0459-9) contains supplementary material, which is available to authorized users.

## Introduction

Diabetic retinopathy (DR) is still a leading cause of blindness in working populations in both developing or advanced countries [1]. Panretinal photocoagulation (PRP) is an established treatment for this serious ocular disease [2], and it has been performed on patients with both severe nonproliferative DR (NPDR) and proliferative DR (PDR) [3]. Diabetes mellitus is a disease that affects the retina and can also cause systemic microcirculatory disorders. When such microvascular complications progress, the risk of developing macroangiopathy also increases [4, 5]. In particular, diabetes impairs the ocular circulation by causing changes in the retinal blood vessels and likely affects choroidal blood flow. There are many unknown factors regarding the effects of DR on choroid and the mechanisms by which PRP changes the choroid.

Since Spaide et al. [6] reported a noninvasive evaluation of choroidal thickness (ChT) in normal volunteers using enhanced depth imaging optical coherence tomography (EDI-OCT) in 2008, several studies on ChT have been reported using EDI method [7–13]. As for ChT in patients with DR, Regatieri and Unsal [7, 8] reported that ChT in patients with diabetic macula edema and PRP-treated PDR were thinner than in normal subjects. In addition, Kim et al. [9] reported that ChT in patients with PDR was significantly increased compared with patients with mild or moderate NPDR, whereas ChT in PRP-treated eyes was thinner.

Several studies [10–13] have reported longitudinal changes in ChT before and after the PRP procedure; however, the results of the studies are still controversial; ChT was found to be increased [10, 11], decreased [12], or increased then decreased [13].

However, measuring ChT in fovea by the pinpoint measurement in EDI is likely influenced by the increase or decrease in the regional ChT as well as the irregularity of the choroidoscleral border [14]. Additionally, measurement of retinal thickness of the central subfield around the fovea provided better reproducibility than did the measurement of the center point [15]. Thus, a 3D volumetric raster scan by swept-source optical coherence tomography (SS-OCT) is considered to be a superior method for measuring the ChT.

The first aim of the study was to clarify the longitudinal ChT changes in PRP-treated eyes. The second aim was to compare ChT among patients with DR as well as normal subjects using SS-OCT.

## Methods

### Patients

Patients with severe NPDR or PDR for whom PRP was considered necessary and performed between August 2014 and March 2015 were enrolled in this study. A total of 70 eyes of 40 patients with severe NPDR and 56 eyes of 36 patients with PDR were enrolled for this study. To compare the severity of DR and ChT, healthy subjects and diabetic patients without DR (noDR), mild to moderate NPDR (m–m NPDR), and severe NPDR and PDR without PRP were enrolled. This study was approved by the Institutional Review Board of Saneikai Tsukazaki Hospital and performed in accordance with the Declaration of Helsinki. Written, informed consent to participate in the study was obtained from all participants. All patients underwent an eye examination including best-corrected visual acuity, slit lamp microscopy, and fundus photography (Optos 200Tx, Optos Inc., Scotland, UK). Visual acuity was presented in logMAR, and the axial length was also measured using IOL Master (Carl Zeiss Meditec, Jena, Germany). Fluorescein angiography (FA) was performed to accurately determine the severity in severe NPDR and PDR eyes. The severity of the retinopathy was based on the classification proposed by the Global Diabetic Retinopathy Project Group [16].

Eyes with a retinal disease other than DR, glaucoma, ocular trauma, history of uveitis, and turbid ocular media that can obscure the border of the choroid in optical coherence tomography (OCT) were excluded. Eyes that had undergone cataract surgery in the last 12 months were also excluded. For eyes with DR, only treatment-naive eyes were included, and those with a history of photocoagulation, vitreous injection of anti-VEGF drugs, and vitreous surgery were excluded. Eyes treated with PRP using a pattern scan laser were excluded.

The right eye was enrolled in the study; if the right eye met any of the exclusion criteria, the left eye was enrolled. For bilaterally PRP-treated eyes with different severity, each eye was enrolled according to its severity.

### Treatment protocol

PRP was performed over four sessions every other week. The procedure was carried out in the following order: (1) superior; (2) nasal; (3) inferior; and (4) temporal. The spot size was set at 200–250 µm with an exposure duration of 0.2–0.3 s. A panfundus contact lens (Ocular Mainster PRP 165, Ocular, Bellevue, WA, USA) was worn, and the total number of exposures was between 900 and 1900. The multicolor photocoagulation laser device (NOVUS Varia, Lumenis, UT, USA) with a wavelength of 532 (green) or 561 (yellow) nm was used. According to the ETDRS protocol [17], the laser power was adjusted to induce gray–white spots.

For eyes that had fovea-involving macular edema before the PRP treatment, or when macular edema was found during treatment, 20-mg sub-Tenon injection of triamcinolone acetonide (STTA) was administered. The decision to treat with STTA was made at the discretion of an experienced ophthalmologist.

### Swept-source optical coherence tomography

The macular area (6 × 6 mm) was measured using SS-OCT (DR1 OCT-1; Topcon, Tokyo, Japan). The SS-OCT system is a swept-source laser device that can provide a scanning speed of 100,000 A-scans per second with a tuning range of approximately 100 nm centered at the 1-µm wavelength region with an imaging depth of 2.6 mm. The SS-OCT measurements were performed by trained examiners after inducing pupil dilation. Using a 3D volumetric raster scan protocol, 3D volumetric data were obtained in 0.8 s, and each 3D scan covered a 6 × 6 mm area centered on the fovea with 512 A-scans × 256 B-scans. To improve the quality of the images, four consecutive B-scan images of the same area were averaged [14].

### Measurements of choroidal thickness

All patients who scheduled for PRP treatment underwent SS-OCT examinations before and after each session at 1, 3, and 6 months after PRP. Additionally, the patients underwent the SS-OCT examination at the time of the initial visit or at the regular visit and their DR severity and ChT were compared. With a set of 64 B-scan images obtained by averaging each of the four consecutive B-scans, a ChT map was created using semiautomatic segmentation. ChT was measured as the distance between the outer border of the retinal pigment epithelium and the inner surface of the choroidal-scleral border. However, semiautomatic segmentation does not always accurately detect the choroidal-scleral border. Therefore, all 64 B-scan images were manually corrected by certified experienced orthoptists using built-in software. The interobserver reproducibility of the ChT measurements obtained by this manual segmentation has previously been reported by our team [14], and the intraclass correlation coefficient (ICC) of the ChT measurements by two observers was extremely favorable (between 0.990 and 0.999).

**Fig. 1:**
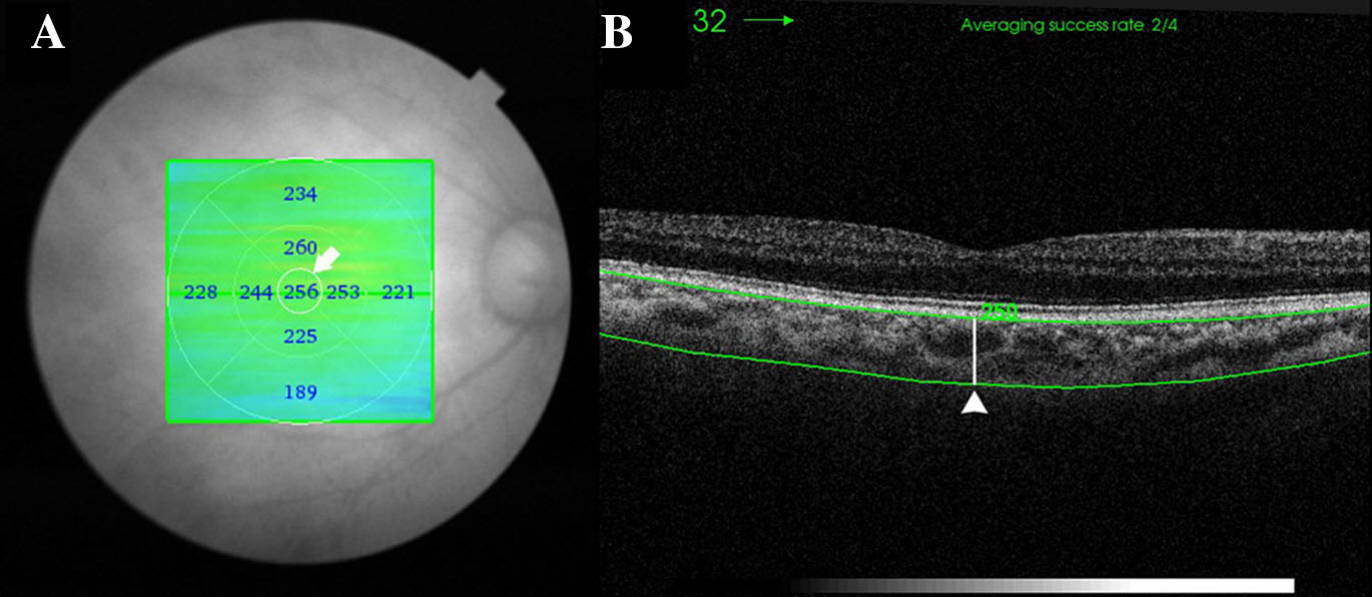
Choroidal thickness (ChT) measurements using SS-OCT. **A** The mean central field choroidal thickness (CFChT) was defined as the mean ChT of the center area spanning a 1 mm diameter on the ETDRS map (*white arrow*). **B** The subfoveal choroidal thickness (SFChT), which is the distance between the outer RPE border and the inner surface of the choroidal–scleral border, was automatically calculated on the images that went through the subfovea (*white arrowhead*)

ChT is automatically divided into sections of the ETDRS map, and the mean ChT of each section is automatically calculated and presented by the software. The inner and outer rings are 3 and 6 mm in diameter, respectively, and each ring is divided into four parts. The mean central field choroidal thickness (CFChT) was defined as the mean ChT of the center area spanning a 1 mm diameter on the ETDRS map (Fig. 1A).

In addition, to obtain the subfoveal choroidal thickness (SFChT) using the same point measurements as the EDI method from the scan images obtained to measure CFChT, the subfovea was manually marked on the images that went through the subfovea. Subsequently, the distance between the outer retinal pigment epithelium border and the inner surface of the choroidal-scleral border was automatically calculated and statistically compared (Fig. 1B).

### Statistical analyses

The statistical analyses were performed using JMP version 10.0 software package (SAS Institute Inc., Cary, NC, USA). A comparison of the duration of DR was performed using a one-way analysis of variance (ANOVA) followed by a Tukey’s HSD test. A comparison of ChT at each time point was performed first by repeated measures one-way ANOVA, and a comparison between groups was performed by Tukey’s HSD test. Additionally, a two-way repeated measures ANOVA was used to compare between STTA-treated and untreated eyes. A *P* value of less than 0.05 was considered statistically significant.

## Results

The characteristics of the PRP-treated patients are presented in Table 1. Ninety-four eyes of 53 patients were excluded as they did not meet the inclusion criteria. Finally, 32 eyes from 23 subjects (6 females and 17 males) completed the study and data were subsequently analyzed.

**Table 1:** Patient demographics in panretinal photocoagulation study

Number of patients (sex)	23 (6 females, 17 males)
Number of eyes	32
Age (years)	55.9 (12.8) (range: 37–79)
Number of eyes by severity level	PDR, 19; severe NPDR, 13
Visual acuity at baseline	0.06 (0.19)
Visual acuity at 6 months	0.09 (0.23)
Axial length, mm	23.6 (0.98)
Mean known disease duration	12.8 (11.5) (range: 1–42)
HbA1c (%)	8.1 (2.1)

Values are shown as mean (SD)

Visual acuity; mean logMAR visual acuity

The mean age (SD) of the PRP patients was 55.6 (±12.8) years (range: 37–79 years). The mean axial length (SD) was 23.6 (±0.98) mm (range: 21.7–26.0 mm), and the mean known disease duration of diabetes mellitus (SD) was 13.5 (±11.8) years (range: 1–43 years). Based on the international classification of DR, 19 eyes were considered PDR and 13 eyes were severe NPDR. The mean (SD) logMAR visual acuity at baseline was 0.06 (±0.19) (range: −0.18 to 0.52), and the logMAR visual acuity at 6 months was 0.09 (±0.23) (range: −0.18 to 0.82).

## Longitudinal ChT changes in PRP

**Fig. 2:**
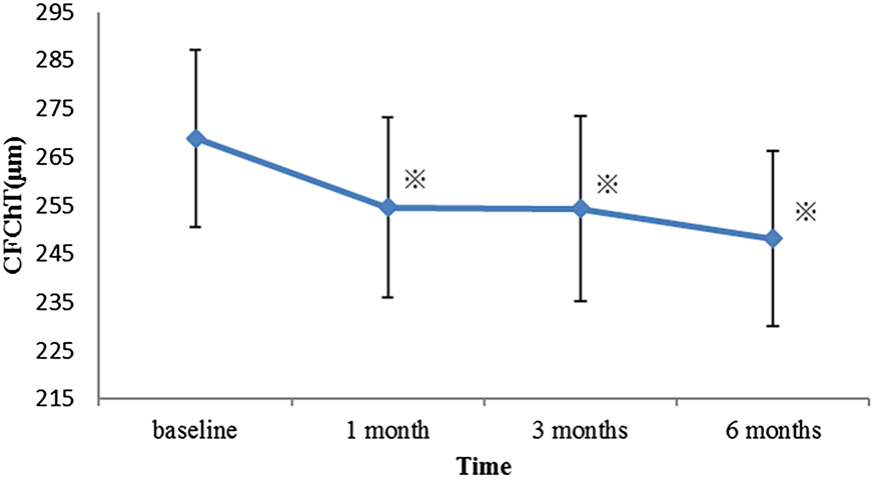
Changes in the central field choroidal thickness (CFChT) after panretinal photocoagulation (PRP). The mean CFChT after PRP at 1, 3, and 6 months was clearly decreased compared with the baseline values. *Asterisk* indicates the significant difference between the measurements points by Tukey’s HSD (*P* < 0.005). *Error bars* represent standard error of the mean

CFChT before treatment was 268.6 ± 104.3 µm and at 1, 3, and 6 months was 254.5 ± 105.3, 254.2 ± 108.2, and 248.1 ± 101.8 µm, respectively. The result of the repeated measures one-way ANOVA was *P* < 0.0001, and the group comparison using a Tukey’s HSD test showed that the mean CFChT was significantly decreased at all post-treatment time points compared with the pretreatment value (all *P* < 0.005) (Fig. 2).

Multiple comparisons tests showed no significant difference between 1 and 3 months and between 3 and 6 months (*P* = 0.9998 and 0.1392, respectively). Moreover, six eyes received STTA during the study period. No statistically significant difference was found for the mean CFChT between the STTA-treated eyes (*N* = 6) and eyes without STTA treatment (*N* = 26) (multivariate ANOVA, *P* = 0.7921).

**Fig. 3:**
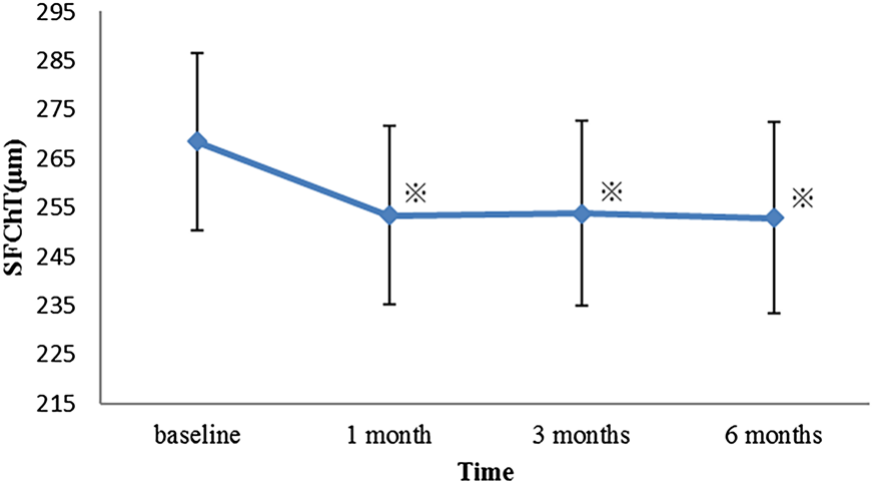
Changes in the subfoveal choroidal thickness (SFChT) after panretinal photocoagulation (PRP). The mean SFChT after PRP at 1, 3, and 6 months was clearly decreased compared with the baseline values. *Asterisk* indicates the significant difference between the measurements points by Tukey’s HSD (*P* < 0.005). *Error bars* represent standard error of the mean

SFChT was 268.4 ± 102.9 µm before treatment and 253.4 ± 103.1, 253.8 ± 107.1, and 252.9 ± 110.5 µm at 1, 3, and 6 months after treatment, respectively. The results of the repeated measures one-way ANOVA were *P* = 0.0002, and the group comparison using the Tukey’s HSD test showed that the mean SFChT was significantly decreased at all post-treatment time points compared with the pretreatment value (all *P* < 0.005) (Fig. 3).

Multiple comparisons tests showed no significant difference between 1 and 3 months and between 3 and 6 months (*P* = 0.9998 and 0.9532, respectively).

## Comparison of ChT among the different groups of DR severity and normal subjects

Table 2 shows the characteristics of the patients included in the comparison of ChT with severity of DR. The mean CFChT of the eyes for each severity matched for the mean age and axial length with the untreated PDR patients was as follows: normal, 248.3 ± 70.7; noDR, 250.2 ± 55.4; m–m NPDR, 230.0 ± 70.3; severe NPDR, 323.2 ± 61.3; and PDR, 307.3 ± 84.1.

**Table 2:** Characteristics of normal subjects and patients with diabetes mellitus by severity

	Normal	noDR	m–m NPDR	Severe NPDR	PDR
Number	20	14	16	11	18
Sex (female)	8 (12)	9 (5)	11 (5)	6 (5)	12 (6)
Age (years)	50.6 (9.4)	50.7 (7.1)	50.6 (7.7)	50.7 (5.7)	50.6 (9.9)
Axial length (mm)	23.8 (0.7)	23.8 (1.2)	23.7 (1.2)	23.6 (1.1)	23.7 (0.9)

Values are shown as mean (SD)

**Fig. 4:**
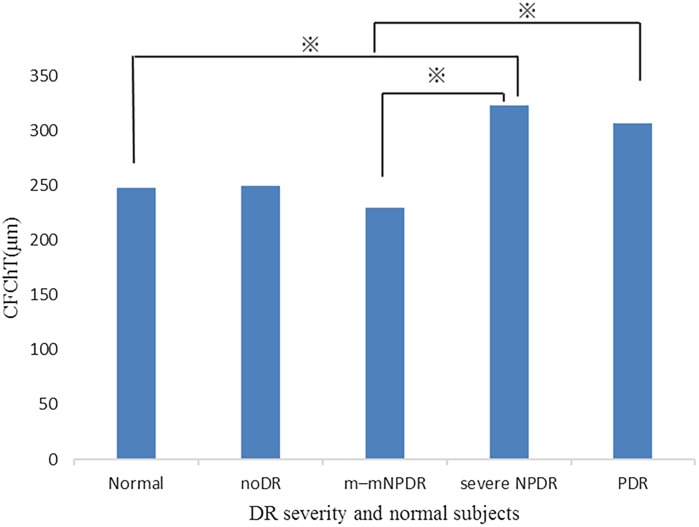
Comparisons of choroidal thickness (ChT) among DR severity groups and normal subjects. *Asterisk* indicates statistically significant pair by Tukey’s HSD test (*P* < 0.005)

ChT of the PDR group was significantly thicker than that of the m–m NPDR group. Moreover, CFChT of the severe NPDR group was significantly thicker than that of the normal and m–m NPDR groups (*P* < 0.005) (Fig. 4).

**Fig. 5:**
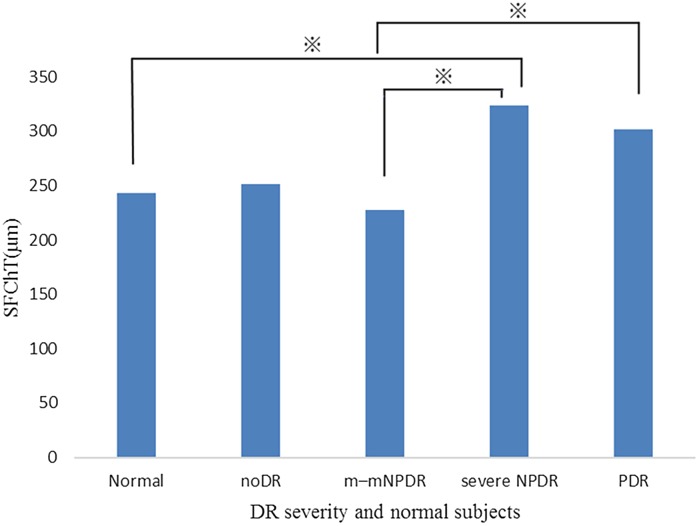
Comparisons of SFChT among DR severity groups and normal subjects. *Asterisk* indicates statistically significant pair by Tukey’s HSD test (*P* < 0.005)

The mean SFChT of the eyes for each severity group was as follows: normal, 243 ± 71.4; noDR, 251.3 ± 61.9; m–m NPDR, 227.1 ± 71.3; severe NPDR, 323.1 ± 66.0; and PDR, 301.7 ± 80.8. In addition, SFChT of the PDR group was significantly thicker than that of the m–m NPDR group; SFChT of the severe NPDR group was significantly thicker than that of the normal and m–m NPDR groups (Fig. 5). The results of CFChT and SFChT were extremely similar.

## Discussion

In the current study, we confirmed that PRP treatment causes a decrease in ChT, which continues for up to 6 months. As photocoagulation scarring becomes enlarged over time [18], changes in ChT are also likely to continue over a long period. The choroid consists of abundant vascular components and may be greatly influenced by changes in its blood flow. Our observation period of 6 months is appropriate because ocular blood flow velocities are stable from 6 months to 2 years following PRP [19].

According to the early longitudinal studies on ChT after PRP [10–13], SFChT increased after 1 week [10, 13]. Cho et al. [10] speculated that increases in ChT are due to increased blood flow due to vasodilation or choroidal effusion induced by the laser, which damages the peripheral choriocapillaris, consequently decreasing the peripheral choroidal blood flow. As a result, redistribution of blood flow occurs, and the choroidal blood flowing around the foveal centralia increases, resulting in an increase in SFChT. However, in the present study, ChT at 1 week after PRP was not measured and thus cannot be directly compared.

Meanwhile, some reports showed that SFChT was decreased at 1 month [12, 13] and 3 months after PRP [13], which suggested that ChT tends to decrease over time. Considering these findings together with the data from our present study, although ChT may increase in the short term, it tends to decrease over the long term.

Zhang et al. [13] presented three hypotheses regarding the observed decreases in ChT: (1) thermal damage by photocoagulation spreads to the choroid which consequently interferes with choroidal reperfusion or reorganization, and results in a reduction in ChT; (2) PRP damages the RPE and decreases VEGF secretion which results in decreased dilation and permeability of the choroidal vessels; (3) the outer retina is destroyed and the hypoxic inner retina comes closer to the highly saturated choriocapillaris, which then improves the hypoxic state. Autoregulation decreases the choroidal blood flow and causes a decrease in ChT.

However, Zhu et al. [11] reported a significant increase in SFChT at 1 and 3 months after PRP, revealing completely different results from other studies. They argued [11] that a thinner ChT around the photocoagulated area causes a reduction in the peripheral choroidal blood flow, resulting in a redistribution of blood supply to the fovea that consequently increases SFChT. In our study as well as others, a conventional laser was used to perform PRP. In contrast, Zhu et al. [11] used a pattern scan laser PRP. It has been reported that the laser scarring from a pattern scan laser reduces rather than expands in size, and the reduction can be up to 35% [20]. In addition, a study which histopathologically compared the scars by both a conventional and pattern scan laser reported that conventional laser scar lesions exhibited both outer and inner nuclear layer loss. In contrast, pattern scan laser scar lesions exhibited only outer nuclear layer loss, and the inner nuclear layer was preserved [21]. This difference may have contributed to the inconsistent results between these studies.

Previous studies have reported that ChT negatively correlates with age and axial length [22, 23]. Therefore, in this study, the mean CFChT was statistically compared with the data matched with PDR patients for age and axial length. The results indicate that the mean ChT of the severe NPDR and PDR groups was significantly thicker than that of the m–m NPDR group. Moreover, the mean ChT of the severe NPDR group was also significantly thicker than that of the normal group. These findings illustrate that as the severity worsens, ChT also increases. These findings are supported by Kim et al. [9], who found that ChT significantly increased as the severity worsened from mild/moderate NPDR to PDR. We suspected that the same point measurements as EDI-OCT might be easily affected by the local increase or decrease in ChT, as well as the irregularity of the choroidoscleral border; however, the values were almost identical to the mean values obtained within the 1-mm center area (CFChT). As the choroid is thickest at the fovea centralis in DR [7–9], this seems to be the reason why there was no difference observed between CFChT and SFChT.

Increases in ChT as the severity of retinopathy worsens are likely caused by an increase in vascular endothelial growth factor (VEGF). DR causes retinal nonperfusion, and VEGF is produced. VEGF is induced by hypoxia and results in elevated intraocular VEGF levels [24–26]. One study found that ChT was thinner when patients were treated with a combination of laser treatment and anti-VEGF therapy than when they were treated with laser treatment alone [27]. Such findings suggest that VEGF causes the choroid to thicken.

As ChT tends to increase the severity of DR and PRP significantly decreases ChT, a positive correlation between ChT and DR activity or intraocular VEGF level may exist. Based on this assumption, the decrease in ChT following PRP and the continued decrease for at least 6 months observed in this study may be associated with a decline in the retinopathy symptoms. Therefore, ChT may be a useful indicator for controlling the progression of DR.

Due to the small sample size of our study, the possibility of biased results cannot be ruled out. The reliability of our results should be further validated by studying a larger number of cases. In our study, there were no statistical differences in ChT between the STTA-treated eyes and those that did not receive treatment; however, there was a study which reported a decrease in ChT in DME eyes following intravitreal triamcinolone acetonide [28]. Thus, this may have influenced our study results. Additionally, having a longer follow-up period may help to clarify the relationship between ChT and severity of retinopathy.

## Conclusion

ChT significantly decreased following PRP, and the decline continued for at least 6 months after treatment. ChT of patients with severe NPDR and PDR was significantly thicker than that with m–m NPDR patients. ChT of patients with DR changed according to the treatment and severity of DR.

## Electronic supplementary material

Below is the link to the electronic supplementary material.

Supplementary material 1 (XLSX 16 kb)

Supplementary material 2 (XLSX 34 kb)
